# New Orleans Medical and Surgical Journal

**Published:** 1848-10

**Authors:** 


*New Orleans Medical and Surgical Journal.*—Drs. Fenner and Carpenter have withdrawn from the editorial corps conducting the above journal, leaving Drs. Harrison & Hester the remaining editors. The New Orleans Journal has been edited with great spirit and ability, and as the retiring editors are to continue their labors as correspondents, we hope its character and usefulness will not suffer by the change. We believe that in general, periodicals flourish best under a monarchical administration. It being understood that, as in other kinds of autocracy, the wisdom of the executive is chiefly exhibited in the success with which he avails him of the talents and labors of others.

